# Compared Binding Properties between Resveratrol and Other Polyphenols to Plasmatic Albumin: Consequences for the Health Protecting Effect of Dietary Plant Microcomponents

**DOI:** 10.3390/molecules191117066

**Published:** 2014-10-24

**Authors:** Norbert Latruffe, Matthias Menzel, Dominique Delmas, René Buchet, Allan Lançon

**Affiliations:** 1 Laboratoire de Biochimie (Bio-peroxIL n°7270), 6 boulevard Gabriel, Université de Bourgogne, Dijon F-21000, France; 2 Centre de Recherche Inserm U866, Université de Bourgogne, Dijon F-21000, France; 3 ICBMS UMR-CNRS 5246, UFR Chimie-Biochimie, Université Claude Bernard, Lyon I, Villeurbanne F-69622, France

**Keywords:** resveratrol, quercetin, fluorescence, affinity, structural changes

## Abstract

Phytophenols are considered to have beneficial effects towards human physiology. They are food microcomponents with potent chemopreventive properties towards the most three frequent contemporary human diseases, e.g., cardiovascular alterations, cancer and neurodegenerative pathologies. Related to this, the plasmatic form and plasmatic level of plant polyphenols in the body circulation are crucial for their efficiency. Thus, determinations of the binding process of resveratrol and of common flavonoids produced by major edible plants, berries and fruits to plasma proteins are essential. The interactions between resveratrol and albumin, a major plasma protein, were compared with those already published, involving curcumin, genistein, quercetin and other well-known food-containing polyphenols. The approaches used are usually intrinsic fluorescence intensity changes, quenching of protein intrinsic fluorescence and infrared spectroscopy. It appears that: (1) all of the studied polyphenols interact with albumin; (2) while most of the studied polyphenols interact at one albumin binding site, there are two different types of resveratrol binding sites for bovine serum albumin, one with the highest affinity (apparent K_D_ of 4 µM) with a stoichiometry of one per monomer and a second with a lower affinity (apparent K_D_ of 20 µM) with also a stoichiometry of one per monomer; (3) at least one binding site is in the vicinity of one tryptophanyl residue of bovine serum albumin; and (4) resveratrol binding to bovine serum albumin produces a very small structural conformation change of the polypeptide chain. These results support a role played by polyphenols-albumin interactions in the plasma for the bio-activities of these food microcomponents in the body.

## 1. Introduction

Numerous studies have reported interesting properties of *trans*-resveratrol as a preventive agent against several important pathologies; vascular diseases, cancers, viral infection and neurodegenerative processes (for reviews, see [1,2,3,4]). In addition, resveratrol may also increase lifespan [5]. Moreover, several epidemiological studies (in particular [6]) revealed that resveratrol would be one of the main wine microcomponents giving benefits to health with moderate wine consumption. On the other hand, the antiproliferative effect of resveratrol has been shown *in vitro*, in several cell lines derived from tumors; for instance, the human hepatoblastoma-derived HepG2 cell line [7] or the SW480 cancer colorectal cell line [8,9]. So far, the important question of the transport and delivery of resveratrol through the body is not completely answered yet. According to its low water solubility [10], resveratrol is preferentially bound to proteins in order to remain at a certain serum concentration as much as possible. On the other hand, the efficiency of a therapeutic substance (including natural bio-active molecules) is related to its capacity (selectivity and affinity) to bind protein transporters [11]. Since albumin, a major plasma protein, is well known to bind to and carry out a large number of amphiphilic molecules, it appears to be a good candidate as a resveratrol plasmatic carrier. To determine the nature of the interaction between resveratrol and albumin (binding sites and binding constants), the methodologies are as follows: fluorescence intensity enhancement [12] by albumin, the quenching of albumin tryptophanyl residues fluorescence and the infrared spectra of resveratrol and albumin.

**Figure 1:**
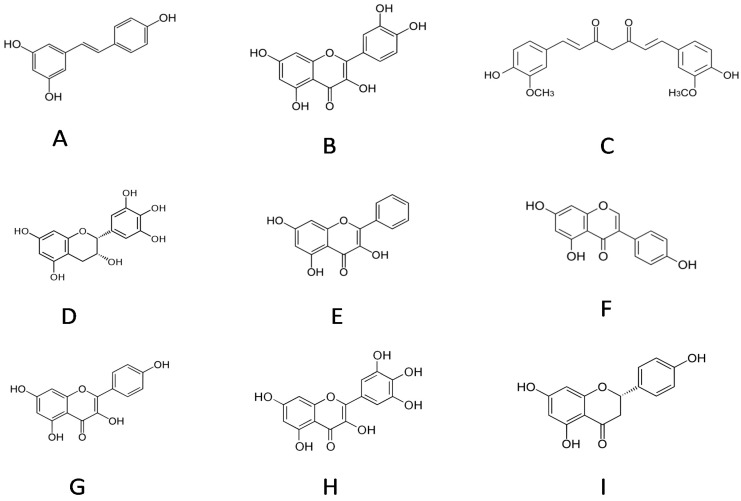
(**A**) resveratrol; (**B**) quercetin; (**C**) curcumin; (**D**) epigallocatechin; (**E**) galangin; (**F**) genistein; (**G**) kaempferol; (**H**) myricetin; (**I**) naringenin.

This review collects the data on the molecular interaction of resveratrol with albumin that contributes to the transport of resveratrol through the blood circulation and that is essential for its further delivery at the cell surface and, consequently, membrane uptake [13] to finally produce its intracellular biological effect. The data on resveratrol are compared to those published concerning albumin interactions with curcumin, genistein, quercetin and other well-known food-containing polyphenols, which may compete with resveratrol to modulate the bio-availabilities of dietary phytophenols (Figure 1).

## 2. Results and Discussion

### 2.1. Binding of Resveratrol to Albumin Followed by Resveratrol and by Protein Tryptophanyl Fluorescences

An increase of resveratrol fluorescence differential with an increasing amount of resveratrol at a given concentration of BSA was previously observed [14], indicating a direct interaction between resveratrol and BSA. Moreover, there is a decrease of the intrinsic fluorescence of BSA tryptophanyl (residues Trp 134 and Trp 213) after adding resveratrol to the medium. The attenuation is resveratrol concentration dependent and reaches 50% with 3 µM of resveratrol, *i.e*., twice more than the BSA concentration (1.5 µM) and 70% of the quenching for a ratio of one to six. The biphasic curve is in agreement with a resveratrol interaction with the two tryptophanyl residues of the albumin polypeptides chain. This quenching has been treated using the representation of Stern-Volmer to determine the number of binding sites and K_D_ (Table 1).

**Table 1:** Data for the resveratrol binding obtained by the attenuation method.

Albumin Form	Concentration (µM)	Scatchard Analysis				Stern Volmer Analysis	
		Type 1 Sites Number (n)	K_D_ Type 1 (µM)	Type 2 Sites Number (n)	K_D_ Type 2 (µM)	K_D_ Type 2 (µM)	FluorophoresNumber
Free FA	0.1	0.8	0.02	1	0.05	0.06	2
Free FA	1	0.8	0.6	1	1.44	1.76	2
Free FA	1.5	0.6	4.8	1.2	7.69	12.71	2
Free FA	3	1.1	4.8	-	-	16.23	2
Fraction V	1	1.1	1.16	2.5	16.7	21.6	2

### 2.2. Determination of Binding Site Number and Dissociation Constants of Resveratrol to Albumin

A Scatchard plot [15] was used to determine the number of resveratrol molecules per binding site and the number of binding sites. Resveratrol binds BSA at two different binding sites, *i.e*., a first one with *n* = 4 per tetramer (one per monomer) and a second, with *n* close to three per tetramer, *i.e*., close to one per monomer, giving a total of sites *n* = 7 per tetramer (theoretically assumed to be *n* = 8, *i.e*., two per monomer) bearing two affinity sites with distinct binding constants (Table 1). Data obtained using the representation of Stern-Volmer and confirmed the existence of two binding sites with a stoichiometry of one for each site per monomer, except in the presence of bound fatty acid to albumin, where the stoichiometry is doubled for the Type 2 site. The apparent dissociation constants (K_D_) show an average value of 4 µM and near 20 µM for high and low affinity sites, respectively.

### 2.3. Resveratrol-Albumin Interactions Observed by Infrared Spectroscopy

**Figure 2:**
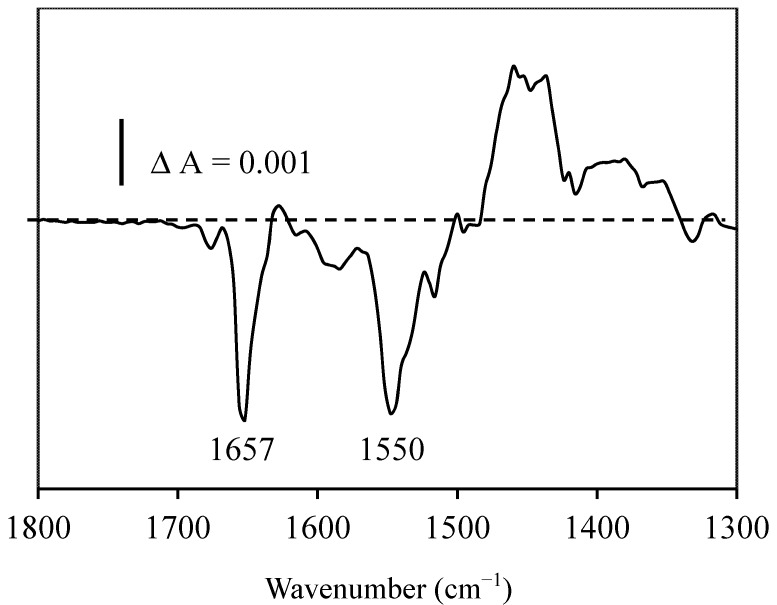
Infrared differential spectrum of resveratrol with BSA (the spectrum of *trans* resveratrol with BSA minus the spectrum of *cis* resveratrol with BSA) obtained after photo-isomerization in the same infrared cell.

The infrared spectra of BSA with or without resveratrol were almost similar (within a 2% experimental error) indicating no-significant secondary structure changes. Therefore, we used reaction-induced infrared difference spectroscopy to better determine the degree of structural changes. The differential IR spectrum of resveratrol (the spectrum of *cis*-resveratrol after photo-isomerization of trans-resveratrol minus the spectrum of *trans*-resveratrol before photo-isomerization) in the presence of BSA (Figure 2) indicates a slight decrease in the intensity of the 1657 cm^−1^ amide-I band. Although the 1657 cm^−1^ band can be assigned to α-helix structures [16,17,18], the intensity change is relatively small (around 0.8%–1.2%, corresponding to four to seven carbonyl groups using a molar absorption coefficient of 2–4 mg^−1^·mL·cm^−1^) and may correspond rather to a slight alteration of the peptide backbone of BSA than to a decrease in the α-helix structure. Indeed, the changes are too small to be assigned solely to the α-helix.

Another change was observed near 1550 cm^−1^, which was assigned mainly to NH groups of the peptide backbone, which became more accessible to ^2^H_2_O and/or which was structurally affected. These results suggest that the *trans-cis* isomerization of resveratrol induces changes in the peptide backbone of BSA within an α-helix and that at least one of the resveratrol isomer (possibly *trans*) interacted with BSA.

Wavenumber (cm^−1^)

### 2.4. Discussion

#### 2.4.1. Resveratrol Interactions with BSA or HAS

We have previously shown that resveratrol is largely bound to serum proteins present in cell culture medium and that *trans*-resveratrol binds to BSA where fatty acids would have a positive effect on this binding [14]. The role of fatty acids should be to ensure a lipophilic environment favorable to the binding of resveratrol. It is also known that the shape of the protein is modified by the presence of fatty acids [19,20]. In the past, molecular interactions of serum albumin with small molecules have been extensively studied [21,22]. We have also shown that cell uptake of a radiolabel is strongly reduced by the presence of bovine serum albumin [13]. Proteins other than albumin may also be implicated in the fixation of resveratrol with higher affinity. For instance, Urpí-Sardà *et al*. [23] have reported binding of resveratrol to human LDL. On the other hand, resveratrol can interact with neuropeptides [24]. Our data established three points: (1) resveratrol interacts with BSA, a major serum protein; (2) there are two resveratrol binding site types in BSA, one with a high affinity (apparent K_D_ of 4 µM or apparent K_A_ of 25 × 10^4^ M^−1^) with a stoichiometry of one per monomer and a second one with a low affinity (apparent K_D_ of 20 µM or apparent K_A_ of 5 × 10^4^ M^−1^) with also a stoichiometry of one per monomer; (3) at least one binding site is located in a pocket containing at least one tryptophanyl residue of BSA; and (4) resveratrol binding to BSA induces a slight structural change of the peptide backbone of BSA associated with an α-helix or distortions of an α-helix. Previously Pantusa *et al*. (2002) [25] measured the intrinsic fluorescence of both human serum albumin (HSA) and resveratrol and showed that resveratrol binds to HSA with an association constant K_A_ of 1.10 ± 0.14 × 10^5^ M^−1^ and reported the formation of an equimolar protein/resveratrol complex (one binding site per monomer), while N’Soukpoe-Kossi *et al.*, 2006 [26], found an association constant K_A_ of 2.56 × 10^5^ M^−1^. Bourassa *et al*. (2010) [27] reported a resveratrol-BSA ratio of *n* = 1.30 with a binding constant K_A_ of 2.52 (±0.5) × 10^4^ M^−1^ which is lower than our values. The differences are probably due to the fact that there are low and high affinity binding sites of resveratrol in BSA.

We showed that resveratrol at 0.5 mM did not significantly alter BSA conformation as inspected from the infrared spectrum of BSA and that of the mixture of BSA with resveratrol, which were similar despite the authors’ claim of a major reduction of α-helix and an increase in the β-sheet [27]. From our opinion, their curve fitting analysis overestimated α-helix content in BSA as compared with that in the mixture of resveratrol and BSA. By using CD spectroscopy, Lu *et al*. (2007) [28] indicated that the secondary structures of HSA and hemoglobin changed in the presence of resveratrol, at a drug to proteins molar ratio of two, with a decrease of the α-helices by 18.75% for HSA and 9.43% for hemoglobin. This is in contrast with N’Soukpoe-Kossi *et al.*, 2006 [26], who reported that at 0.125 mM resveratrol concentrations, no structural changes were observed during resveratrol binding to has, whereas at 1 mM resveratrol, an increase of the α-helix from 57% to 62% and a decrease of the β-sheet from 10% to 7% occurred in the resveratrol-HSA complexes. In our opinion, at the concentration range of 0.125 to 0.5 mM of resveratrol, the secondary structure changes induced by resveratrol binding to either BSA or HSA are too small to be reliably detected by classical infrared spectra (see the review [17] for a critical analysis of secondary structures by infrared spectroscopy) or by CD. The reaction-induced infrared difference spectrum (RIDS) is able to detect very small IR changes produced by a chemical reaction in the same infrared cell (in this case, it was the *trans*-*cis* isomerization of resveratrol induced by photolysis in the same cell keeping exactly the same protein concentrations before and after photolysis). RIDS is around ten-times more sensitive, inducing around 0.1% sample to sample errors compared to classical infrared spectroscopy inducing 1%–2% sample to sample errors [29]. Our RIDS (Figure 2) indicated the difference in binding between *trans* and *cis* resveratrol to BSA. Either *trans* or *cis* resveratrol or both isomers binding to BSA induced a small peptide backbone distortion involving four to seven carbonyl groups of the peptide backbone. Human albumin can stabilize the biologically effective *trans* form [30], suggesting that it is rather the *trans* resveratrol that binds to serum proteins. Taken together, these findings show that the secondary structure alterations caused by *trans* resveratrol binding to BSA or HSA are very small. At a higher resveratrol concentration (1 mM), the reported experimental data [26] need to be reevaluated. The binding of resveratrol by serum proteins allows its transport in the blood and facilitates its accessibility to the cell surface. On the opposite side, the binding affinity should not be too high in order to allow reversibility and resveratrol delivery.

#### 2.4.2. Comparisons with Other Dietary Polyphenol, such as Quercetin

Dawra * et al.*, 1988 [31], have determined the protein binding capacities of ellagic acid and quercetin. They correspond to 297.3 µg bovine serum albumin (BSA)/mg and 78.0 µg BSA/mg, respectively. Sengupta and Sengupta, 2002 [32], studied the binding of quercetin to human serum albumin (HSA), which leads to a strong enhancement in the fluorescence emission intensity and anisotropy, along with significant changes in the fluorescence excitation and emission profiles. The excitation spectrum suggests the occurrence of efficient Förster-type resonance energy transfer from the single tryptophan-214 residue of HSA to the protein bound to quercetin. Furthermore, they calculated the binding constant (K_A_ = 1.9 × 10^5^ M^−1^) and the Gibbs free energy change (ΔG = −30.12 kJ/mol)) for the quercetin-HSA interaction. In 2003, Sengupta and Sengupta [33] also reported two different binding sites of HSA to quercetin based on the steady-state emission spectra. Far-UV CD spectroscopy revealed that binding of quercetin does not induces significant perturbation in the secondary structure of HSA. Kitson, 2004 [34], deduced that the binding to BSA at pH 7.4 was maximal when the quercetin concentration was 10 µM. They postulated that the binding site of BSA for quercetin was less available at higher protein concentrations, perhaps because of conformational change or self-association. Papadopoulou *et al.*, 2005 [35], indicated that quercetin has a total quenching effect on BSA tryptophan fluorescence at a molar ratio of 10:1 (quercetin:BSA), while rutin quenched at approximately a 25:1 molar ratio. In contrast, epicatechin and catechin did not fully quench tryptophan fluorescence over such a molar ratio. Furthermore, their data suggested that the association between flavonoids and BSA did not change the molecular conformation of BSA. Quenching constants were determined using the Stern–Volmer equation to provide a measure of the binding affinity between the flavonoids and BSA. The binding affinity was strongest for quercetin and ranked in the decreasing order: quercetin > rutin > epicatechin = catechin. Interestingly, Kaldas *et al.*, 2005 [36], claimed that quercetin in the presence of peroxidase/hydrogen peroxide covalently links to proteins with a particularly high affinity for HSA, which also may also occur *in vivo*. Dufour and Dangles, 2005 [37], considered that flavonoids display moderate affinities for albumins (binding constants in the range of (1–15) × 10^4^ M^−1^), where flavones and flavonols are tightly bound. Glycosidation and sulfation decrease the affinity to albumin by one order of magnitude, depending on the conjugation site. Despite multiple binding sites of quercetin to albumin, it is proposed that the binding of flavonols primarily takes place in subdomain IIA of albumin [37]. Rolinski *et al.*, 2007 [38], showed in HSA a high sensitivity of tryptophanyl residue (Trp 214) fluorescence to binding quercetin. By estimating the values of the affinity indexes and of the thermodynamic equilibrium constants, Martini *et al.*, 2008 [39], suggested that there is a much stronger capacity of quercetin to interact with BSA when compared with the quercetin conjugated form (the 3-*O*-beta-D-glucopyranoside derivative).

#### 2.4.3. Comparisons with Flavonoids, such as Curcumin, Epigallocatechin, Galangin, Genistein, Kaempferol, Myricetin and Naringenin

Xiao *et al.* (2008) [40] reported that the B-ring hydroxylation of flavonols significantly affects the binding/quenching process, where the binding affinity is generally increased with the number of hydroxyl groups on the B-ring. The binding constants (K_A_) are the following: myricetin (4.90 × 10^8^ M^−1^) > quercetin (3.65 × 10^7^ M^−1^) > kaempferol (2.57 × 10^6^ M^−1^) > galangin (6.43 × 10^5^ M^−1^). The glycoside substitute at the C-ring position decreased the binding affinity. Bourassa *et al*. (2010) [28] showed that the BSA binding stoichiometry (n) for polyphenol is 1.30 for genistein (Gen) and 1.0 for curcumin (Cur), and the polyphenol-BSA binding constants were: K_A_ (Gen-BSA) = 1.26 (±0.3) × 10^4^ M^−1^, and K_A_ (Cur-BSA) = 3.33 (±0.8) × 10^4^ M^−1^. Fluorescence emission spectrometry and molecular docking were applied by Skrt *et al.* (2012) [41] to compare the experimentally determined binding parameters of polyphenols with molecular modeling. Among these polyphenols, (−)-epicatechin-3-gallate showed the highest Stern–Volmer modified quenching constant, followed by (−)-epigallocatechin-3-gallate. Similarly, (−)-epicatechin-3-gallate had the highest effect on the CD spectrum of BSA, while the changes induced by other polyphenols were negligible. Molecular docking predicted high binding energies for (−)-epicatechin-3-gallate and (−)-epigallocatechin-3-gallate for the binding site on BSA near Trp 213. Concerning polyphenol interactions, Kim *et al.*, 2012 [42], found that quercetin inhibited the binding of curcumin to albumin and increased the uptake of curcumin by 60% into WiDr cell line from human colon carcinoma. A summary on binding sites and binding constants of resveratrol and of dietary flavonoids to plasmatic albumin and dependent polypeptide chain conformational changes is presented in the Table 2. It appears that there is a broad range in the binding constant according to the type of molecule. For the same compound, a large disparity is observed with the different reports. Concerning resveratrol, this paper is the only one to have shown two different binding sites on albumin with distinct affinity constants. Sengupta and Sengupta (2003) have also reported two different binding sites of HSA to quercetin.

**Table 2:** Comparison of binding site number and binding constants of dietary polyphenols to plasmatic albumin.

Polyphenol	Type 1 Sites Number (n)	K_D_ Type 1 (µM)	Type 2 Sites Number (n)	K_D_ Type 2 (µM)	Ref.
Resveratrol	1	4 ± 0.4	2	20 ± 2	This paper (Menzel, 2014)
Resveratrol	1.3	253 ± 50			[27]
Resveratrol		25			[26]
Curcumin	1	333 ± 80			[27]
Genistein	1.3	126 ± 30			[27]
Quercetin		0.36			[40]
Quercetin	Yes	19	Yes	?	[32,33]
Myricetin		0.049			[40]

## 3. Materials and Methods

### 3.1. Chemicals

*Trans*-resveratrol, fatty acid-free BSA (bovine serum albumin) and fatty acid-containing BSA (Fraction V) were purchased from Sigma (St. Louis, MO, USA). Methanol and ethanol were purchased from Merck (Darmstadt, Germany) and from Carlo Erba (Milano, Italy), respectively. The chemicals and solutions used in the cell culture were from Gibco BRL (Life Technologies, Scotland, UK). Fetal bovine serum was obtained from Biochrom KG (Berlin, Germany). *Trans*-resveratrol was solubilized in ethanol. All solutions containing resveratrol were wrapped in aluminum foil for protection against light and stored at 4 °C when not used.

### 3.2. Fluorescence

Absorbance measurements were carried out with a Kontron UVIKON spectrophotometer and fluorescence emission with a Kontron SFM 25 spectrofluorometer. For absorbance higher than 0.4 units, correction of the “internal filter effect” (IFE), leading to an attenuation of the fluorescence intensity, was performed according to [14]. The stoichiometry of ligand binding and ligand affinity constants was determined according to Scatchard [15] and adapted for the fluorescence approach according to Azzi [43]. Multisite analysis was done according to Dahlquist [44] and Levine [29].

### 3.3. Infrared Spectra

All solutions containing *trans* resveratrol in DMSO were wrapped in aluminum foil for protection against light and stored at 4 °C when not used. These were mixed with 10 mg·mL^−1^ of BSA in deuterated water and 100 mM Tris HCl at pH corresponding to deuterated solution *i.e.*, p^2^H = 7.8, in the dark (the total DMSO concentration was 5% v:v). A 20-μL sample was loaded between two CaF_2_ windows of a demounTable 25 °C thermostated cell (model Harrick) separated with a 50-μm Teflon spacer. The infrared spectra of BSA and of resveratrol in the dark (*trans* resveratrol isomer spectrum) or after 5 min of UV light illumination (150-W xenon lamp without a filter) (*cis* resveratrol isomer spectrum) were measured using a Nicolet 510 M Infrared spectrometer (Nicolet) equipped with a DTGS detector (Detector of Tri-Glycine Sulfate); 128 interferograms were recorded at a 4-cm^−1^ optical resolution. Three independent measurements were performed, and spectra were co-added and averaged.

## 4. Concluding Remarks

**Figure 3:**
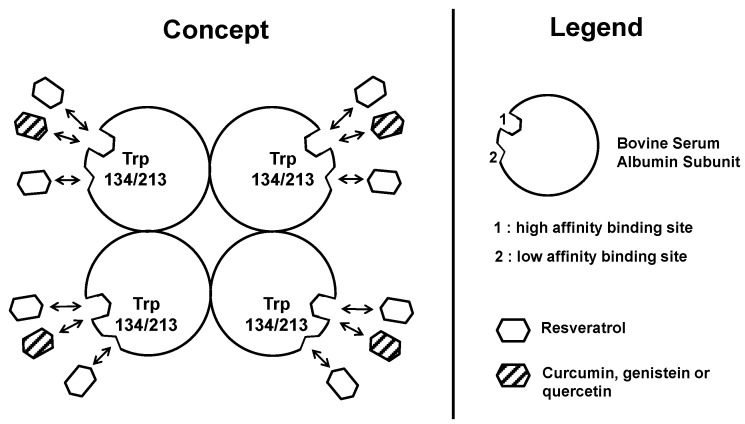
Proposed competitive interaction between resveratrol and other dietary polyphenols toward albumin. Consequences for their bioavailability.

Figure 3 proposes the competitive interaction between resveratrol and other dietary polyphenols toward albumin. According to the binding site number and the affinity constant values summarized in Table 2, it appears that, in the plasma, resveratrol would be bound to albumin preferably over other polyphenols absorbed during a meal. This would explain, in part, the well-known bio-activity of resveratrol despite its relatively low level in the diet, for instance compared to quercetin, a major plant polyphenol.
